# Effectiveness of low-intensity atorvastatin 5 mg and ezetimibe 10 mg combination therapy compared with moderate-intensity atorvastatin 10 mg monotherapy: A randomized, double-blinded, multi-center, phase III study

**DOI:** 10.1097/MD.0000000000036122

**Published:** 2023-11-24

**Authors:** Seung-Ah Lee, Soon Jun Hong, Jung-Hoon Sung, Kyung-Soo Kim, Seong Hwan Kim, Jin Man Cho, Sung Wan Chun, Sang Rok Lee, Chul Sik Kim, Tae Nyun Kim, Dae Hyeok Kim, Hwan-Cheol Park, Byung Jin Kim, Hyun-Sook Kim, Ji-Yong Choi, Young Joon Hong, Joong Wha Chung, Seong Bo Yoon, Sang-Hak Lee, Cheol Whan Lee

**Affiliations:** a Department of Medicine, Asan Medical Center, University of Ulsan College of Medicine, Seoul, Republic of Korea; b Cardiovascular Center, Department of Cardiology, Korea University Anam Hospital, Seoul, Korea; c Department of Cardiology, CHA Bundang Medical Center, CHA University, Seongnam, Republic of Korea; d Division of Endocrinology and Metabolism, Department of Internal Medicine, CHA Bundang Medical Center, CHA University School of Medicine, Seongnam, Republic of Korea; e Department of Cardiology, Korea University Ansan Hospital, Ansan, Republic of Korea; f Cardiovascular Center, Kyunghee University Hospital at Gangdong, Seoul, Republic of Korea; g Department of Endocrinology, Soonchunhyang University College of Medicine, Cheonan, Republic of Korea; h Division of Cardiology, Jeonbuk National University Hospital, Jeonju, Republic of Korea; i Division of Endocrinology, Yongin Severance Hospital, Yonsei University College of Medicine, Youngin, Republic of Korea; j Department of Endocrinology and Metabolism, Haundae Paik Hospital, Inje University College of Medicine, Busan, Republic of Korea; k Division of Cardiology, Department of Internal Medicine, Inha University Hospital, Incheon, Republic of Korea; l Division of Cardiology, Department of Internal Medicine, Hanyang University College of Medicine, Cardiovascular center, Hanyang University Guri Hospital, Guri, Republic of Korea; m Division of Cardiology, Department of Internal Medicine, Kangbuk Samsung Hospital, Sungkyunkwan University School of Medicine, Seoul, Republic of Korea; n Department of Cardiology, Hallym University Sacred Heart Hospital, Anyang, Republic of Korea; o Department of Internal Medicine, Daegu Catholic University, Daegu, Republic of Korea; p Department of Cardiology, Chonnam National University Medical School, Gwangju, Republic of Korea; q Department of Internal Medicine, Chosun University Hospital, Gwangju, Republic of Korea; r Department of Cardiology, H-Plus Yangji Hospital, Seoul, Republic of Korea; s Division of Cardiology, Severance Hospital, Yonsei University College of Medicine, Seoul, Republic of Korea.

**Keywords:** atorvastatin, cholesterol, ezetimibe, hypercholesterolemia, LDL

## Abstract

**Background:**

We compared the efficacy and safety of low-intensity atorvastatin and ezetimibe combination therapy with moderate-intensity atorvastatin monotherapy in patients requiring cholesterol-lowering therapy.

**Methods:**

At 19 centers in Korea, 290 patients were randomized to 4 groups: atorvastatin 5 mg and ezetimibe 10 mg (A5E), ezetimibe 10 mg (E), atorvastatin 5 mg (A5), and atorvastatin 10 mg (A10). Clinical and laboratory examinations were performed at baseline, and at 4-week and 8-week follow-ups. The primary endpoint was percentage change from baseline in low-density lipoprotein (LDL) cholesterol levels at the 8-week follow-up. Secondary endpoints included percentage changes from baseline in additional lipid parameters.

**Results:**

Baseline characteristics were similar among the study groups. At the 8-week follow-up, percentage changes in LDL cholesterol levels were significantly greater in the A5E group (49.2%) than in the E (18.7%), A5 (27.9%), and A10 (36.4%) groups. Similar findings were observed regarding the percentage changes in total cholesterol, non-high-density lipoprotein cholesterol, and apolipoprotein B levels. Triglyceride levels were also significantly decreased in the A5E group than in the E group, whereas high-density lipoprotein levels substantially increased in the A5E group than in the E group. In patients with low- and intermediate-cardiovascular risk, 93.3% achieved the target LDL cholesterol levels in the A5E group, 40.0% in the E group, 66.7% in the A5 group, and 92.9% in the A10 group. In addition, 31.4% of patients in the A5E group, 8.1% in E, 9.7% in A5, and 7.3% in the A10 group reached the target levels of both LDL cholesterol < 70 mg/dL and reduction of LDL ≥ 50% from baseline.

**Conclusions:**

The addition of ezetimibe to low-intensity atorvastatin had a greater effect on lowering LDL cholesterol than moderate-intensity atorvastatin alone, offering an effective treatment option for cholesterol management, especially in patients with low and intermediate risks.

## 1. Introduction

Statins are the basis for the treatment of hypercholesterolemia and are generally well tolerated in most patients. However, as with all drugs, some patients cannot tolerate regimens recommended by current guidelines^[1,2]^ and require the reduction of the dose of statin.^[3–5]^ In fact, low-intensity statins are occasionally used in real-world clinical practice, leading to suboptimal control of low-density lipoprotein (LDL) cholesterol levels. Ezetimibe interrupts dietary and biliary cholesterol absorption and lowers LDL cholesterol levels by approximately 15% to 20%.^[6]^ The combination of ezetimibe and statin provides substantial incremental reductions in LDL cholesterol with lesser side effects.^[7]^

Atorvastatin is one of the most widely prescribed statins in the world. Moderate-intensity atorvastatin (10–20 mg) is recommended for reduction of LDL cholesterol levels by 30% to 49% in individuals with low or intermediate risk.^[2]^ Low-intensity atorvastatin (5 mg) monotherapy is considered insufficient in decreasing LDL cholesterol levels by ≥ 30%.^[8]^ Ezetimibe and low-intensity atorvastatin may synergistically lower LDL cholesterol levels with treatment goal achievement.^[7]^ However, little data are available on whether this approach has similar efficacy and fewer side effects compared with moderate-intensity atorvastatin (10 mg). In the present study, we compared the efficacy and safety of a fixed-dose combination of low-intensity atorvastatin 5 mg and ezetimibe 10 mg (A5E) with those of ezetimibe 10 mg (E), low-intensity atorvastatin 5 mg (A5), and moderate-intensity atorvastatin 10 mg (A10) on the lipid profiles of patients requiring cholesterol-lowering therapy.

## 2. Methods

### 2.1. Trial design

This randomized, double-blind, placebo-controlled trial comparing the effects of A5E combination therapy to E, A5, and A10 monotherapy on lipid levels and their safety profiles in patients with hypercholesterolemia was conducted at 19 centers in Korea (ClinicalTrials.gov identifier: NCT05131997). The institutional review board at each site approved the study protocol, and all subjects provided written informed consent.

Patients who met the inclusion/exclusion criteria at Visit 1 received a placebo to check for drug compliance and discontinued lipid medications for at least 4 weeks (4 weeks for statins and omega-3 supplements and 6 weeks for fibrates) (Supplementary Table 1, http://links.lww.com/MD/K731 and Supplementary Fig. 1, http://links.lww.com/MD/K734). The patients were screened based on their fasting serum lipid levels and the inclusion/exclusion criteria at Visit 2. The risk factors and lipid levels were used to determine cardiovascular disease risk category according to the 2018 Korean guideline for the management of dyslipidemia (Supplementary Table 2, http://links.lww.com/MD/K732).^[9]^ Eligible patients were stratified based on the cardiovascular risk category at Visit 2 and randomized to 1 of 4 treatment groups: A5E, E, A5, and A10. The patients visited the research institution every 4 weeks for efficacy and safety evaluations.

### 2.2. Participants

Patients were eligible for enrollment if they had LDL cholesterol fasting serum levels ≤ 250 mg/dL and triglyceride levels < 400 mg/dL at Visit 1 and had appropriate ranges of LDL cholesterol levels according to the defined risk category at Visit 2. Patients who had the following conditions were excluded: a history of acute coronary syndrome, ischemic stroke, percutaneous coronary intervention or coronary bypass graft surgery within the last 6 months; uncontrolled type 2 diabetes (fasting glucose > 160 mg/dL or HbA1c > 9%); uncontrolled hypertension; secondary dyslipidemia; comorbidities such as active liver disease, biliary disease, chronic kidney disease (estimated glomerular filtration rate < 30 mL/min/1.73 m^2^), hyperthyroidism, hypothyroidism, or malignant tumors within the last 5 years; impaired drug absorption such as Crohn disease or ulcerative colitis; a history of fibromyalgia, myopathy, or rhabdomyolysis; and those who are pregnant or are breastfeeding.

### 2.3. Intervention

Randomization was performed individually using a web-based interactive response system. Addpharma Co., Ltd. prepared the investigational drugs according to the randomization list. The investigational drugs (4 pills once a day: one actual medication and 3 placeboes) were prescribed for 8 weeks. The patients and researchers were blinded to the trial-group assignments, and therapeutic lifestyle changes were encouraged throughout the study period.

### 2.4. Endpoints

The primary efficacy endpoint was a percentage change in LDL cholesterol level from baseline to an 8-week follow-up. Secondary efficacy endpoints included changes in the following variables: LDL cholesterol at a 4-week follow-up, and total cholesterol, high-density lipoprotein (HDL) cholesterol, triglyceride, non-HDL cholesterol, apolipoprotein A1, apolipoprotein B, and LDL cholesterol goal achievement at 4- and 8-week follow-up. The treatment goal of LDL cholesterol was individualized according to the risk category (Supplementary Table 2, http://links.lww.com/MD/K732). For safety endpoint, adverse events were monitored throughout the treatment period. Major safety endpoints included adverse events, serious adverse events, and incidences of creatine kinase and liver enzyme elevations.

### 2.5. Statistical analysis

This trial was designed to show the superiority of A5E over E and A5 in percentage reduction of LDL cholesterol levels. A10 as the reference standard of moderate-intensity statin was used to assess the utility of A5E in lowering LDL cholesterol. The full analysis set was used for the efficacy assessment and the safety analysis set for the safety assessment. The full analysis set included patients who had a primary end point assessment at least once after administration of an investigational drug, and the safety analysis set included all those who administered an investigational drug. Efficacy and safety analyses were performed for the intention-to-treat population. Efficacy was established by co-primary endpoints by using a gatekeeping method. If the superiority of A5 over E in the reduction of LDL cholesterol levels is demonstrated, then the superiority of the A5E combination treatment over E and A5 was assessed.

The sample size was calculated to be 240 patients (60 patients per group) to obtain an overall 80% power of test combined with a 20% drop-out assuming a weighted mean difference of −10.43% in LDL cholesterol level in the A5 and E groups with a standard deviation of 14.3% from the conservative perspective.^[8,10–12]^ For the continuous variables, analysis of variance (Kruskal–Wallis test) was used to assess significant differences in the baseline characteristics among the groups and the chi-square test or Fisher exact test was used for categorical variables. The percentage changes in LDL cholesterol and other lipid parameters among the groups were evaluated using the analysis of the mixed effect model for repeated measures. Two-sided tests were performed at a significance level of 0.05 for all statistical analyses in this study. All statistical analyses were conducted using SAS version 9.4 (SAS Institute, Cary, NC).

## 3. Results

### 3.1. Patients characteristics

**Figure 1.**
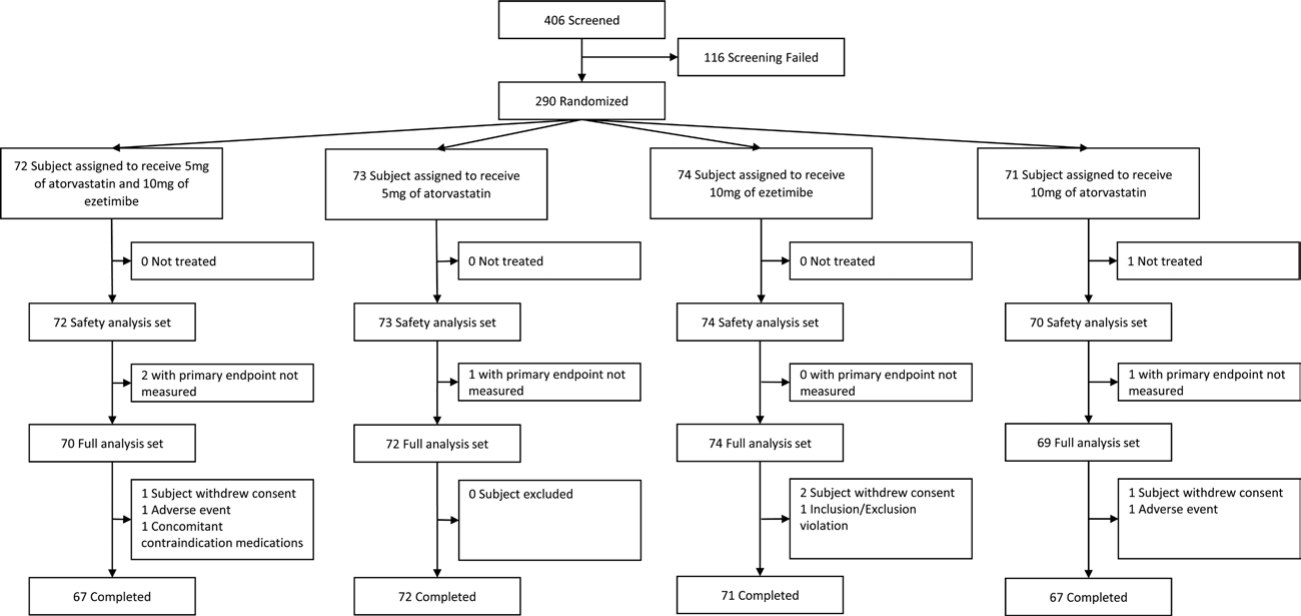
Study flow diagram.

A total of 406 patients were screened, of whom 290 were randomized to one of the 4 treatment groups: A5E (n = 72), E (n = 74), A5 (n = 73), or A10 (n = 71). The first patient was enrolled in November 2021, and the last patient was enrolled in March 2022. All patients received at least one dose of the study drug. A total of 277 patients (95.5%) completed the study while 13 (4.5%) discontinued; 9 (3.1%) withdrew consent, 2 (0.7%) experienced adverse events, and 2 (0.7%) discontinued for other reasons (Fig. 1). Baseline patient characteristics were similar across treatment groups (Table 1). Overall, the median age was 62.0 (interquartile range, 53–68) years, 35.5% were women, 41.7% had type 2 diabetes, 70% had hypertension, and 79.7% belonged to the high- or very high-risk group.

**Table 1:** Baseline characteristics.

Characteristic	Atorvastatin 5 mg/ezetimibe 10 mg (n = 72)	Ezetimibe 10 mg (n = 74)	Atorvastatin 5 mg (n = 73)	Atorvastatin 10 mg (n = 71)
Age, mean (SD), years	60.7 (11.8)	60.7 (11.3)	58.0 (11.5)	59.9 (10.8)
Male sex, n (%)	49 (68.1)	47 (63.5)	44 (60.3)	47 (66.2)
BMI, mean (SD), kg/m^2^	26.6 (3.4)	25.8 (3.3)	25.6 (4.2)	26.2 (3.2)
Risk factors, n (%)				
Current smoker	15 (20.8%)	19 (25.7%)	23 (31.5%)	18 (25.4%)
Diabetes mellitus	34 (47.2%)	28 (37.8%)	29 (39.7%)	30 (42.3%)
Hypertension	51 (70.8%)	47 (63.5%)	55 (75.3%)	50 (70.4%)
Medical history, n (%)				
Asymptomatic CAD	3 (4.2%)	2 (2.7%)	1 (1.4%)	2 (2.8%)
Angina pectoris	18 (25.0%)	25 (33.8%)	27 (37.0%)	22 (31.0%)
Myocardial infarction	5 (6.9%)	8 (10.8%)	4 (5.5%)	4 (5.6%)
Cerebral infarction	1 (1.4%)	0 (0.0%)	3 (4.1%)	3 (4.2%)
Carotid artery stenosis	2 (2.8%)	1 (1.4%)	1 (1.4%)	1 (1.4%)
Peripheral venous disease	0 (0.0%)	0 (0.0%)	0 (0.0%)	1 (1.4%)
Risk category, n (%)				
Low	4 (5.6%)	5 (6.8%)	4 (5.5%)	4 (5.6%)
Moderate	11 (15.3%)	10 (13.5%)	11 (15.1%)	10 (14.1%)
High	23 (31.9%)	20 (27.0%)	22 (30.1%)	16 (22.5%)
Very high	34 (47.2%)	39 (52.7%)	36 (49.3%)	41 (57.8%)

CAD = coronary artery disease, SD = standard deviation.

### 3.2. Efficacy outcomes

**Figure 2.**
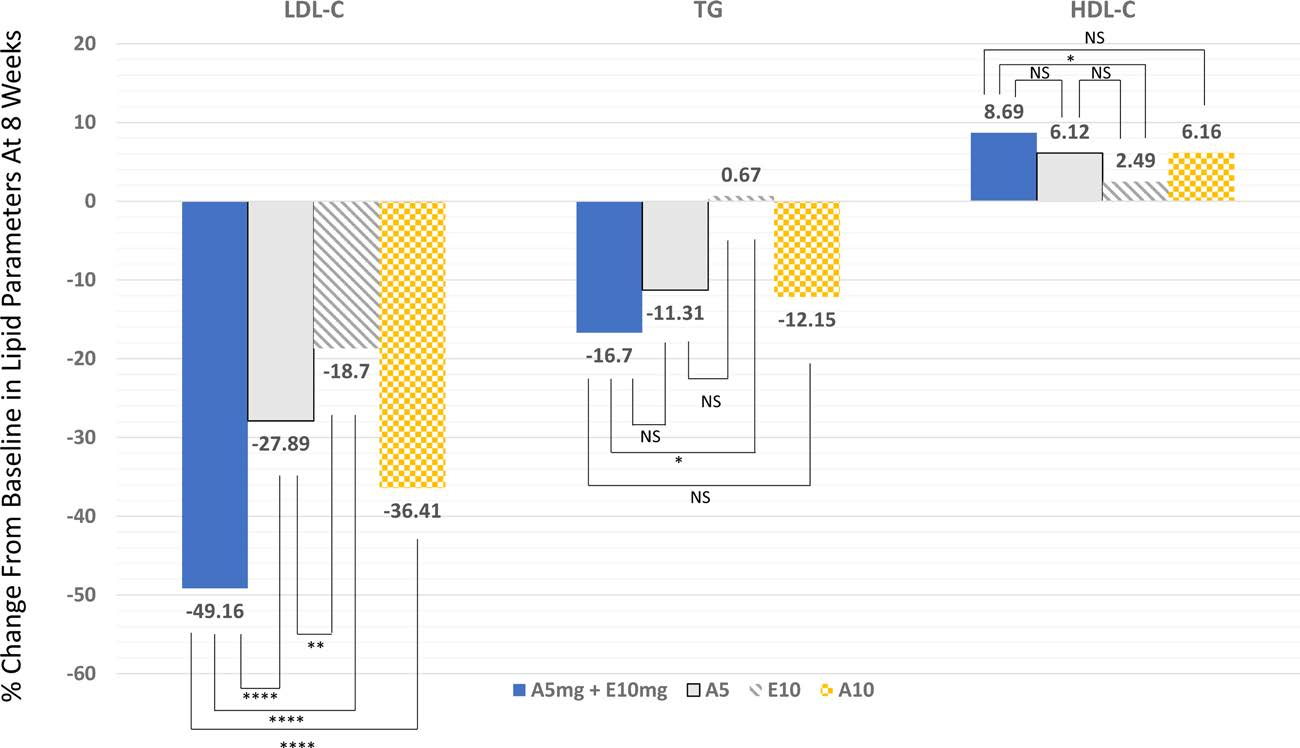
Percentage changes in lipid profile at 8-wk follow-up. A10 = atorvastatin 10 mg, A5 = atorvastatin 5 mg, A5E = atorvastatin 5 mg and ezetimibe 10 mg, E = ezetimibe 10 mg, HDL-C = high-density lipoprotein cholesterol, LDL-C = low-density lipoprotein cholesterol, NS = not significant (*P* ≥ .05), TG = triglyceride. ****P < .0001; ***P < .001; **P < .01; *P < .05.

The efficacy results are presented in Figure 2. At the 8-week follow-up, the A5E group exhibited a significantly higher percentage reduction in LDL cholesterol levels (49.2%) compared to the E group (18.7%, *P* < .0001), the A5 group (27.9%, *P* < .0001), and the A10 group (36.4%, *P* < .0001). These differences were evident at the 4-week follow-up and persisted throughout the study period (Supplementary Fig. 2, http://links.lww.com/MD/K735). Similar findings were observed with regards to the total cholesterol, non-HDL cholesterol, and apolipoprotein B levels (Supplementary Table 3, http://links.lww.com/MD/K733). Interestingly, reductions in triglyceride levels were more significant in the A5E group than in the E group. HDL levels increased significantly in the A5E group than in the E group.

**Figure 3.**
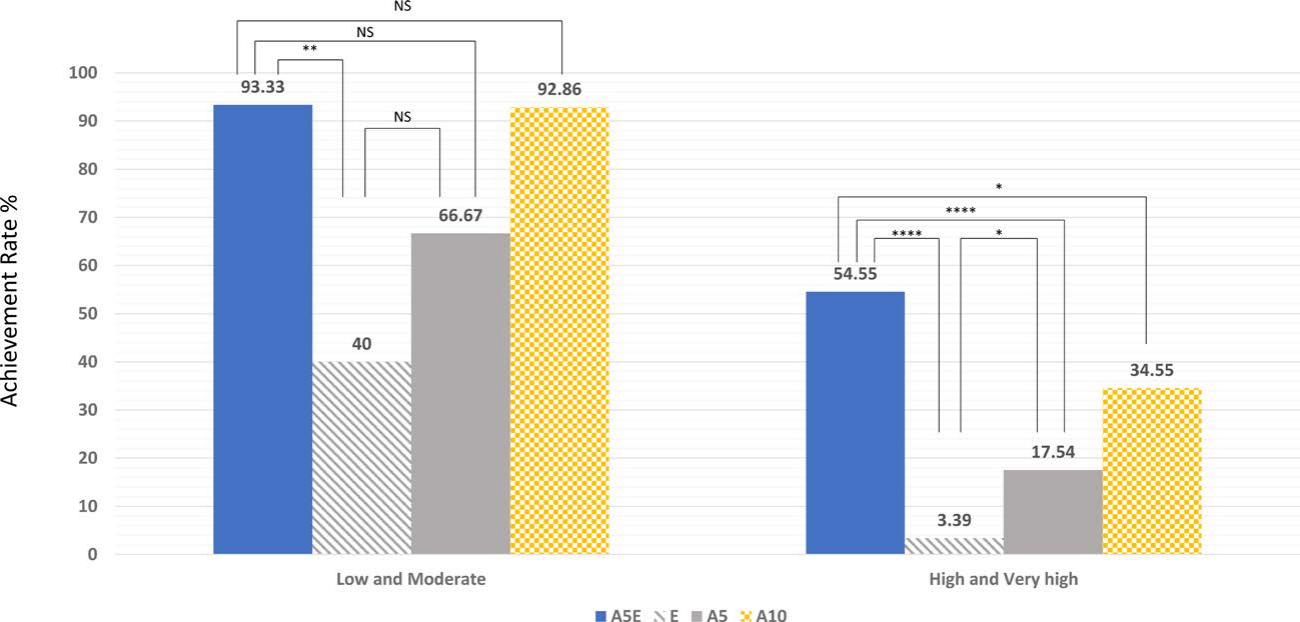
Achievement of target low-density lipoprotein cholesterol goal according to the cardiovascular risk category at 8-wk follow-up. A10 = atorvastatin 10 mg, A5 = atorvastatin 5 mg, A5E = atorvastatin 5 mg and ezetimibe 10 mg, E = ezetimibe 10 mg, NS = not significant (*P* ≥ .05). ****P < .0001; ***P < .001; **P < .01; *P < .05.

Figure 3 shows the proportion of patients who achieved LDL cholesterol targets at the 8-week follow-up according to the 2018 Korean guideline for the management of dyslipidemia. In addition, the attainment of LDL cholesterol goals, as per the 2018 Korean guideline for the management of dyslipidemia and the 2019 ESC/EAS guidelines for the management of dyslipidaemias, categorized into 4 risk groups, is illustrated in Supplementary Figure 3A, http://links.lww.com/MD/K736 and Supplementary Figure 3B, http://links.lww.com/MD/K737. Regarding patients with low and moderate risks, 93.3% of them achieved target LDL cholesterol levels in the A5E group, 40% in the E, 66.7% in A5, and 92.9% in A10. In addition, a higher percentage of patients in the A5E group (31.4%) achieved LDL cholesterol level < 70 mg/dL and reduction of LDL ≥ 50% than those in the E (8.1%), A5 (9.7 %), or A10 (7.3%) groups at week 8 (Supplementary Fig. 4, http://links.lww.com/MD/K738).

### 3.3. Safety outcomes

The overall incidence of treatment-related adverse events was 6.2%, which were similar among all the groups being mild to moderate (Table 2). There were no serious adverse events. Two adverse events led to discontinuation of the study drug in the A5E (n = 1, 1.4%) and A10 (n = 1, 1.4%) groups. An increase in the level of either alanine or aspartate aminotransferase (>3 upper limits of normal) was reported in one of the 72 patients (1.4%) in the A5E group and in 3 of the 70 patients (4.3%) in the A10 group. There were no cases of myopathy or rhabdomyolysis.

**Table 2:** Treatment-related side effects.

Variable	Atorvastatin 5mg/ezetimibe 10 mg (n = 72)	Ezetimibe 10 mg (n = 74)	Atorvastatin 5g (n = 73)	Atorvastatin 10 mg (n = 70)
Adverse drug reaction	7 (9.7)	5 (6.8)	1 (1.4)	5 (7.1)
Mild	5 (6.9)	3 (4.1)	1 (1.4)	4 (5.7)
Moderate	2 (2.8)	2 (2.7)	0 (0)	1 (1.4)
Adverse drug reaction leading to withdrawal	1 (1.4)	0 (0)	0 (0)	1 (1.4)
Reported adverse drug reaction				
Abdominal pain	1 (1.4)	0 (0)	0 (0)	0 (0)
Dyspepsia	0 (0)	0 (0)	0 (0)	1 (1.4)
Liver function tests increased	1 (1.4)	0 (0)	0 (0)	3 (4.3)
Headache	1 (1.4)	0 (0)	0 (0)	0 (0)
Pruritus	1 (1.4)	0 (0)	0 (0)	0 (0)

The values are presented as number (%).

## 4. Discussion

The principal finding of this study is that treatment with low-intensity A5 plus E significantly lowered LDL cholesterol levels by 49.2% over 8 weeks compared with moderate-intensity A10 (36.4%). This difference was accompanied by a higher rate of LDL cholesterol goal achievement (93.3% of patients with low and intermediate risks and 54.6% of those with high and very high risks). The results also showed that A5E had favorable effects on total cholesterol, non-HDL cholesterol, and apolipoprotein B. Adverse events were infrequent and did not differ across the groups. These findings suggest that the strategy of using a fixed-dose combination therapy of low-intensity A5E may be more suited for patients with low and intermediate risks. However, this strategy achieved the LDL cholesterol goal of < *70 mg*/*dL* and ≥ *50*% *reduction* from baseline in only 31.4% of patients, suggesting that it is not sufficient to lower LDL cholesterol levels in patients with high and very high risks.

Current guidelines emphasize the importance of reducing LDL cholesterol levels in patients with atherosclerotic cardiovascular disease (ASCVD) as well as in apparently healthy people. The 2018 American College of Cardiology/American Heart Association guideline recommends high intensity statin for adjunctive lipid-lowering therapy in patients with high and very high risks, and moderate-intensity statin in those with low and intermediate risks.^[2]^ In general, high-intensity statins reduce LDL cholesterol levels by ≥ 50% and moderate-intensity statin by 30% to 49%. In the present study, the low-intensity A5E combination therapy achieved superior LDL cholesterol reduction (49.2% vs 36.4%, *P* < .001) compared with moderate-intensity A10. These findings suggest that this approach is at least equivalent to moderate-intensity statin therapy for decreasing LDL cholesterol and achieving LDL cholesterol target in patients with low and intermediate risks. The present data are also consistent with those of a previous report using low-intensity rosuvastatin 2.5 mg plus E.^[13]^ However, only 54.6% of patients using low-intensity A5E combination therapy achieved ≥ 50% LDL cholesterol reduction, indicating that this approach is not strong enough to improve LDL cholesterol in patients with high and very high risks.

Numerous studies have established the key role of statins in cardiovascular risk reduction in patients with ASCVD, and recommended the intensification of LDL cholesterol-lowering therapy according to their baseline risk. However, statin-related adverse effects are usually dose-dependent, and some patients are intolerant to moderate-intensity statins.^[3–5]^ It is well established that LDL cholesterol is the major atherogenic lipoprotein and the primary target of therapy for ASCVD prevention.^[1,2]^ Numerous epidemiologic and clinical trials have documented a clear relationship between LDL cholesterol levels and ASCVD events.^[14–16]^ Although high- and moderate-intensity statins remain the standard for cholesterol management, adverse effects may limit their wide use. Furthermore, recent studies support that statin/ezetimibe combination therapy improves atherosclerotic plaque inflammation and clinical outcomes similar to statin monotherapy at equivalent LDL cholesterol-lowering doses.^[17,18]^ In this regard, our regimen is considered an attractive alternative to moderate-intensity statins, especially for patients with low and intermediate risks who are intolerant to moderate-intensity statins.

This study has several limitations. First, it was conducted on a small number of patients using surrogate endpoints. There remains uncertainty as to the utility of this approach in reducing clinical events, requiring cardiovascular outcome trials. Second, our approach may not be generalizable to patients with high and very high risks because it had only moderate potency in reducing LDL cholesterol.

## 5. Conclusion

The fixed-dose combination of low-intensity atorvastatin and ezetimibe significantly reduced LDL cholesterol as well as additional lipid parameters than moderate-intensity atorvastatin alone among patients requiring cholesterol-lowering therapy.

## Acknowledgments

Addpharma Co., Ltd. or its representatives designed the trial and provided the investigational drugs. They were responsible for the design and conduct of the trial, and the collection and analysis of the data. They had no role in data interpretation or the writing of the manuscript based on the data.

## Author contributions

**Conceptualization:** Cheol Whan Lee, Sang-Hak Lee.

**Data curation:** Seung-Ah Lee.

**Formal analysis:** Seung-Ah Lee.

**Funding acquisition:** Cheol Whan Lee, Sang-Hak Lee.

**Investigation:** Soon Jun Hong, Jung-Hoon Sung, Kyung-Soo Kim, Seong Hwan Kim, Jin Man Cho, Sung Wan Chun, Sang Rok Lee, Chul Sik Kim, Tae Nyun Kim, Dae Hyeok Kim, Hwan-Cheol Park, Byung Jin Kim, Hyun-Sook Kim, Ji-Yong Choi, Young Joon Hong, Joong Wha Chung, Seong Bo Yoon, Cheol Whan Lee, Sang-Hak Lee.

**Supervision:** Soon Jun Hong, Jung-Hoon Sung, Kyung-Soo Kim, Seong Hwan Kim, Jin Man Cho, Sung Wan Chun, Sang Rok Lee, Chul Sik Kim, Tae Nyun Kim, Dae Hyeok Kim, Hwan-Cheol Park, Byung Jin Kim, Hyun-Sook Kim, Ji-Yong Choi, Young Joon Hong, Joong Wha Chung, Seong Bo Yoon, Cheol Whan Lee, Sang-Hak Lee.

**Writing – original draft:** Seung-Ah Lee.

**Writing – review & editing:** Soon Jun Hong, Jung-Hoon Sung, Kyung-Soo Kim, Seong Hwan Kim, Jin Man Cho, Sung Wan Chun, Sang Rok Lee, Chul Sik Kim, Tae Nyun Kim, Dae Hyeok Kim, Hwan-Cheol Park, Byung Jin Kim, Hyun-Sook Kim, Ji-Yong Choi, Young Joon Hong, Joong Wha Chung, Seong Bo Yoon, Cheol Whan Lee, Sang-Hak Lee.

## Supplementary Material
