# Supplementary material for: Effectiveness of low-intensity atorvastatin 5 mg and ezetimibe 10 mg combination therapy compared with moderate-intensity atorvastatin 10 mg monotherapy: A randomized, double-blinded, multi-center, phase III study
Source: Medicine (Baltimore). 2023 Nov 24;102(47):e36122. doi: 10.1097/MD.0000000000036122 (PMC10681377; doi:10.1097/MD.0000000000036122)
Supplement: Supplementary file 1 [file medi-102-e36122-s001.docx]

**Supplementary Table 1. Inclusion and exclusion criteria**

**Inclusion criteria**

**At Visit 1**

1. Patients aged ≥19 years
2. Patients with primary hypercholesterolemia
3. Patients with the following fasting serum lipid measurements at Visit 1:
4. LDL-C ≤250mg/dL
5. Triglycerides < 400 mg/dL
6. Patients who voluntarily provided written consent to participate in this clinical trial

**At Visit 2**

1. Patients who met the criteria (Korean Guidelines for the Management of Dyslipidemia 4th edition) in each group by checking the fasting serum lipid measurements (LDL-C and TG) according to cardiovascular risk factors at the time of randomization
   - The risk factors below the table are reference material for classification based on the Korean Guidelines for the Management of Dyslipidemia 4th edition. They are not items for evaluating the selection/exclusion criteria.

| **Risk category** | **Risk Factors**^*^ | **LDL-C**^a^ **(mg/dL)** | **TG**^b^**(mg/dL)** |
| --- | --- | --- | --- |
| Low-risk group (Group1) | at least 1 of the risk factors (See below^†^) | 160-250 | <400 |
| Moderate-risk group (Group2) | at least 2 of the risk factors (See below^†^) | 130-250 | <400 |
| High-risk group (Group3) | Carotid artery disease (when significant carotid artery stenosis is confirmed), Abdominal aortic aneurysm, Diabetes | 100-250 | <400 |
| Very high-risk group (Group3) | Coronary artery disease, Atherosclerotic ischemic stroke and transient cerebral ischemic attack, Peripheral artery disease | 70-250 | <400 |

^*^ Based on the Korean Guidelines for the Management of Dyslipidemia 4th edition

^a^ LDL-C, low-density lipoprotein cholesterol

^b^ TG, Triglycerides

^†^ The major risk of cardiovascular diseases except LDL-cholesterol

- Age: male ≥ 45 years, female ≥ 55 years
- Familial early onset of coronary artery disease: When coronary artery disease occurs in a parent or sibling (male < 55 years, female < 65 years)
- Hypertension: SBP ≥140 mmHg or DBP ≥90 mmHg or taking antihypertensive drugs
- Smoking: continuous smoking
- Low HDL-C: HDL-C < 40 mg/dL
- High HDL-C: If HDL-C ≥ 60mg/dL, it is considered a protective factor and one is subtracted from the total number of risk factors.

1. Patients who met the appropriate criteria when reconfirming inclusion/exclusion criteria at the time of randomization (however, criteria applicable only to screening are excluded)

**Exclusion criteria**

1. Excluded diseases
   1. Patients with secondary dyslipidemia
   2. Patients with uncontrolled hypertension (SBP ≥180 mmHg or DBP ≥110 mmHg)
   3. Patients who were diagnosed with one or more of following medical history within the last 6 months from the Visit 1
      1. Patients with ischemic heart disease (unstable angina, myocardial infarction), peripheral artery disease, or a history of percutaneous transvascular coronary artery dilatation or coronary artery bypass surgery, etc.
      2. Patients with cerebrovascular diseases (cerebrovascular disease, cerebral infarction, cerebral hemorrhage, transient cerebral ischemia, etc.)
2. Medical history and comorbidities
   1. Patients with a history of gastrointestinal diseases (Crohn's disease, ulcers, etc.) and surgeries (except simple appendectomy or hernia surgery) that may affect drug absorption, distribution, metabolism, and excretion
   2. Patients with a medical or family history of fibromyalgia, myopathy, rhabdomyolysis, or hereditary myopathy or patients with a history of HMG-CoA reductase inhibitor- or fibrate-induced muscular toxicity
   3. Patients with active liver disease and severe liver impairment
   4. Patients with atresia of bile ducts or cholestasis
   5. Patients with a diagnosis of malignancy, including leukemia and lymphoma, within 5 years prior to visit 1 (Patients who have been evaluated as a complete response after treatment and who have not relapsed within at least 2 years from the visit 1 or those whose malignancy is the only basal cell carcinoma or squamous cell carcinoma of the skin can be enrolled)
3. Clinical laboratory test results
   1. Patients with serum ALT and AST levels 3 times the upper limit of normal
   2. Patients with CK(CPK) levels more than 3 times higher than the upper limit of normal
   3. Patients with SCr levels more than twice higher than the upper limit of normal
   4. Patients with eGFR < 30 mL/min/1.73 m^2^
   5. Patients with uncontrolled type diabetes (HbA1c > 9.0% or Fasting blood sugar > 160mg/dL)
   6. Patients with uncontrolled hyperthyroidism or hypothyroidism (patients with TSH levels 1.5 times higher than the upper limit of normal)
4. History of allergy and hypersensitivity
   1. Patients resistant or hypersensitive to the HMG-CoA reductase inhibitor and Ezetimibe or to the ingredients of the two drugs
   2. Patients with hereditary conditions such as galactose intolerance, Lapp lactase deficiency, or glucose-galactose malabsorption
5. Contraindicated treatment and drug therapy

Patients who are taking drugs that are contraindicated in combination, such as glecaprevir and pibrentasvir, or are expected to take them during the clinical trial period

1. Others
   1. Patients with a history of substance or alcohol abuse within 1 year before Visit 1
   2. Pregnant or breastfeeding female patients or patients positive for a pregnancy test
   3. If the female patients or the male partner's female patients is a woman of childbearing potential who has not undergone sterilization, patients who do not agree to use the following contraceptive methods during the clinical trial period
      1. For both female and partner of male patients: combination of intrauterine device/tubal ligation and sterilization/condom or diaphragm and spermicide
      2. Male patients (not applicable to female subjects' male partners): Condoms
   4. Patients administered a different investigational drug within 3 months before Visit 2(If the investigational drug was not administered or of the patients participated in a non-interventional observational study, enrollment of possible)
   5. Patients who did not maintain a washout period and at least 4 weeks of lead-in (for Fibrates, 6 weeks of washout and 4 weeks or more of lead-in) at the time of randomization)
2. Patients deemed ineligible for participation in the clinical trial based on clinical findings by the investigators
