# Supplementary material for: Effectiveness of low-intensity atorvastatin 5 mg and ezetimibe 10 mg combination therapy compared with moderate-intensity atorvastatin 10 mg monotherapy: A randomized, double-blinded, multi-center, phase III study
Source: Medicine (Baltimore). 2023 Nov 24;102(47):e36122. doi: 10.1097/MD.0000000000036122 (PMC10681377; doi:10.1097/MD.0000000000036122)
Supplement: Supplementary file 2 [file medi-102-e36122-s002.pptx]

## Slide 1
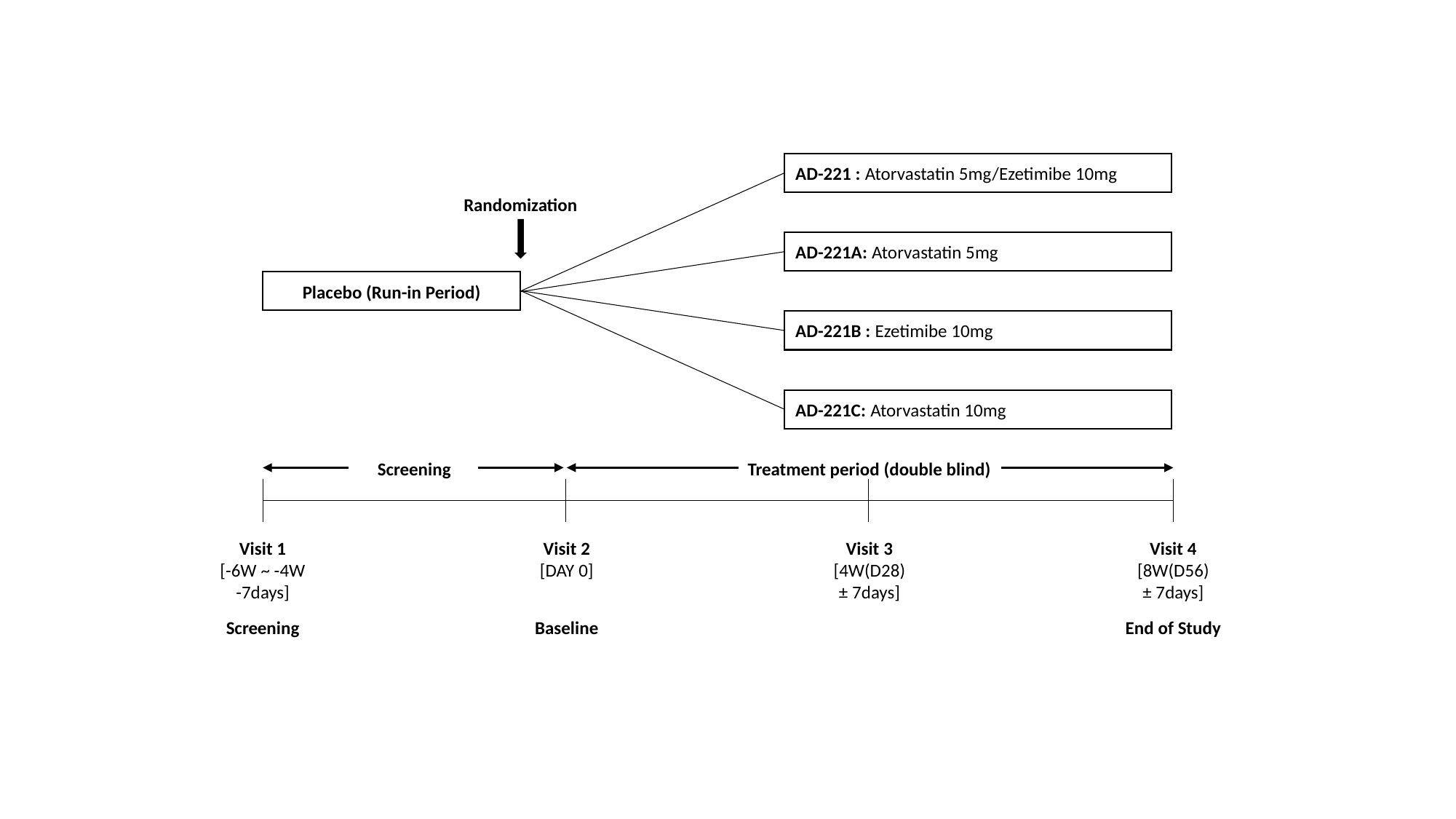

AD-221 : Atorvastatin 5mg/Ezetimibe 10mg
AD-221A: Atorvastatin 5mg
AD-221B : Ezetimibe 10mg
AD-221C: Atorvastatin 10mg
Randomization
Placebo (Run-in Period)
Screening
Treatment period (double blind)
Visit 1
[-6W ~ -4W
-7days]
Visit 2
[DAY 0]
Visit 3
[4W(D28)
± 7days]
Visit 4
[8W(D56)
± 7days]
Screening
Baseline
End of Study
