# Supplementary material for: Effectiveness of low-intensity atorvastatin 5 mg and ezetimibe 10 mg combination therapy compared with moderate-intensity atorvastatin 10 mg monotherapy: A randomized, double-blinded, multi-center, phase III study
Source: Medicine (Baltimore). 2023 Nov 24;102(47):e36122. doi: 10.1097/MD.0000000000036122 (PMC10681377; doi:10.1097/MD.0000000000036122)
Supplement: Supplementary file 3 [file medi-102-e36122-s003.docx]

**Supplementary Table 2.** Risk category and LDL cholesterol goals

| **Risk category** | **Risk Factors**^*^ | **LDL**^a^ **cholesterol goals (mg/dL)** |
| --- | --- | --- |
| Low-risk | at least 1 of the risk factors (See below^†^) | <160 |
| Moderate-risk group | at least 2 of the risk factors (See below^†^) | <130 |
| High-risk group | Carotid artery disease (when significant carotid artery stenosis is confirmed), Abdominal aortic aneurysm, Diabetes | <100 |
| Very high-risk group | Coronary artery disease, Atherosclerotic ischemic stroke and transient cerebral ischemic attack, Peripheral artery disease | <70 |

^*^ based on the Korean Guidelines for the management of dyslipidemia 4th edition

^a^ LDL = low-density lipoprotein

^†^ The major risk of cardiovascular diseases except LDL-cholesterol

- Age: male ≥ 45 years, female ≥ 55 years
- Familial early onset of coronary artery disease: When coronary artery disease occurs in a parent or sibling (male < 55 years, female < 65 years)
- Hypertension: systolic bold pressure ≥140 mmHg or diastolic blood pressure ≥90 mmHg or taking antihypertensive drugs
- Smoking: continuous smoking
- Low HDL (high-density lipoprotein) cholesterol: HDL cholesterol < 40 mg/dL
- High HDL cholesterol: If HDL cholesterol ≥ 60mg/dL, it is considered a protective factor and one is subtracted

from the total number of risk factors.
