# Supplementary figures and images for: Effectiveness of low-intensity atorvastatin 5 mg and ezetimibe 10 mg combination therapy compared with moderate-intensity atorvastatin 10 mg monotherapy: A randomized, double-blinded, multi-center, phase III study
Source: Medicine (Baltimore). 2023 Nov 24;102(47):e36122. doi: 10.1097/MD.0000000000036122 (PMC10681377; doi:10.1097/MD.0000000000036122)

## Slide 1
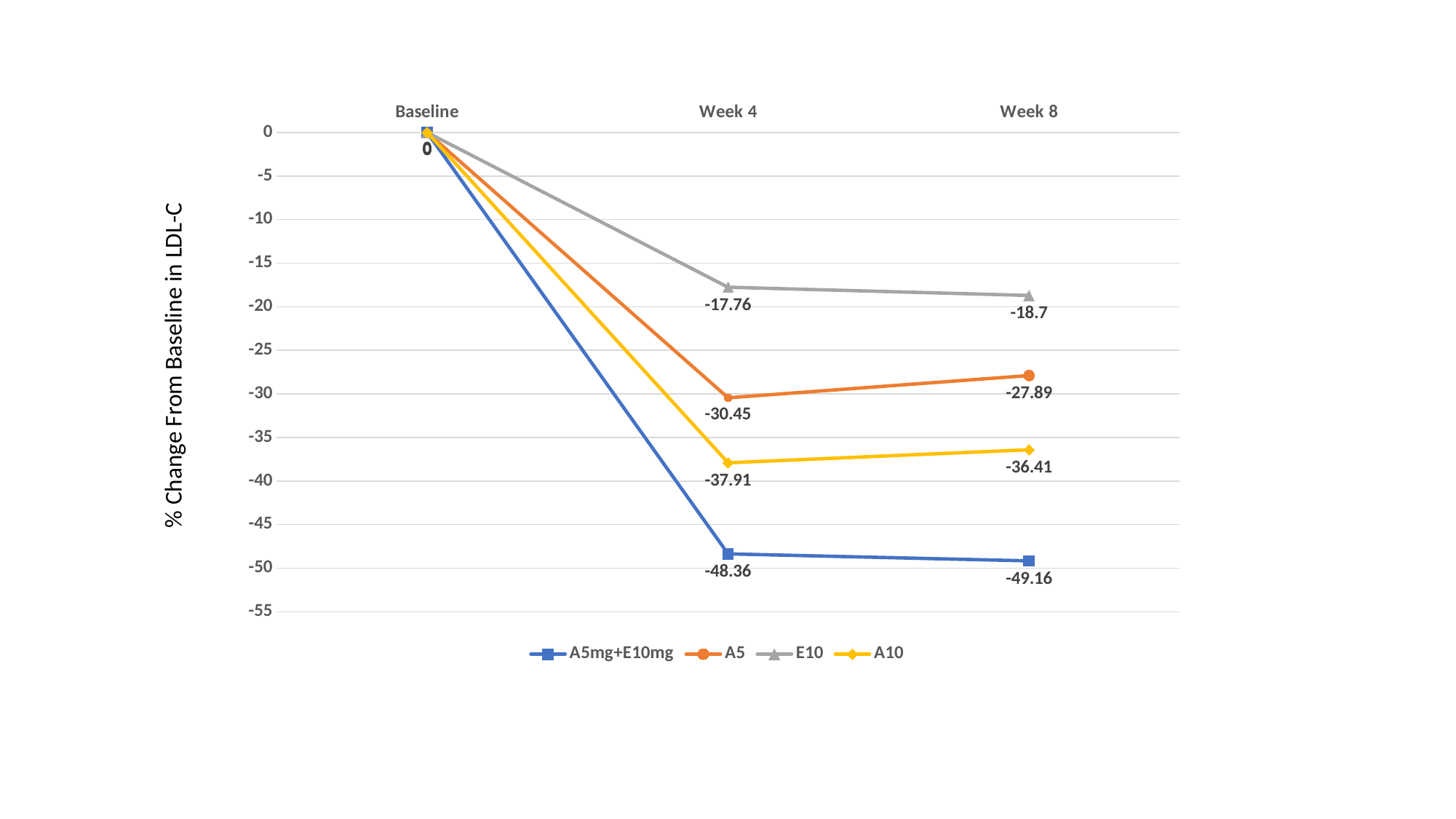

### Chart
| Category | A5mg+E10mg | A5 | E10 | A10 |
|---|---|---|---|---|
| Baseline | 0.0 | 0.0 | 0.0 | 0.0 |
| Week 4 | -48.36 | -30.45 | -17.76 | -37.91 |
| Week 8 | -49.16 | -27.89 | -18.7 | -36.41 |% Change From Baseline in LDL-C

Supplement: Supplementary file 4 [file medi-102-e36122-s004.pptx]

## Slide 1
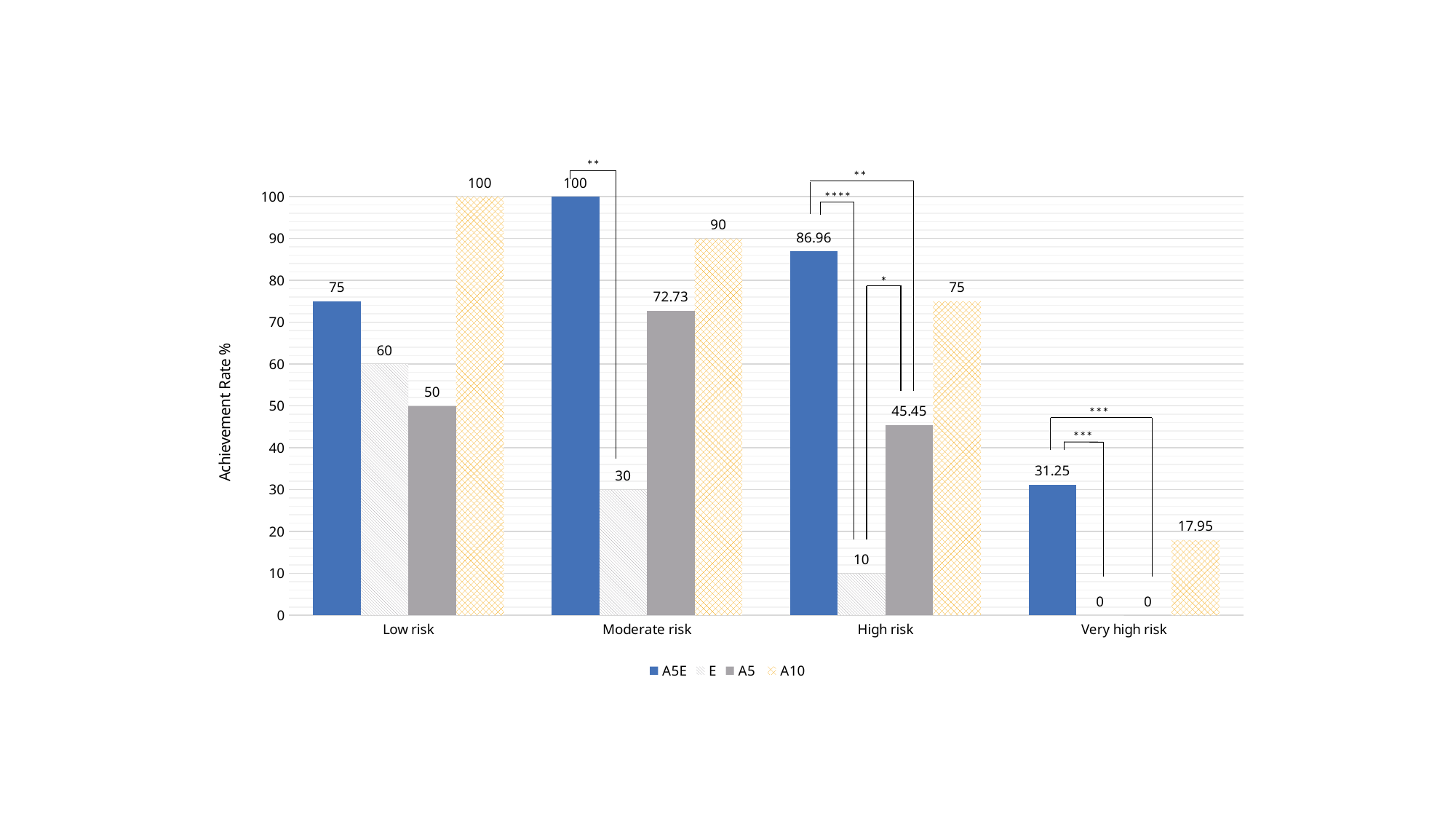

**
**
### Chart
| Category | A5E | E | A5 | A10 |
|---|---|---|---|---|
| Low risk | 75.0 | 60.0 | 50.0 | 100.0 |
| Moderate risk | 100.0 | 30.0 | 72.73 | 90.0 |
| High risk | 86.96 | 10.0 | 45.45 | 75.0 |
| Very high risk | 31.25 | 0.0 | 0.0 | 17.95 |****
*
***
***

Supplement: Supplementary file 6 [file medi-102-e36122-s006.pptx]

## Slide 1
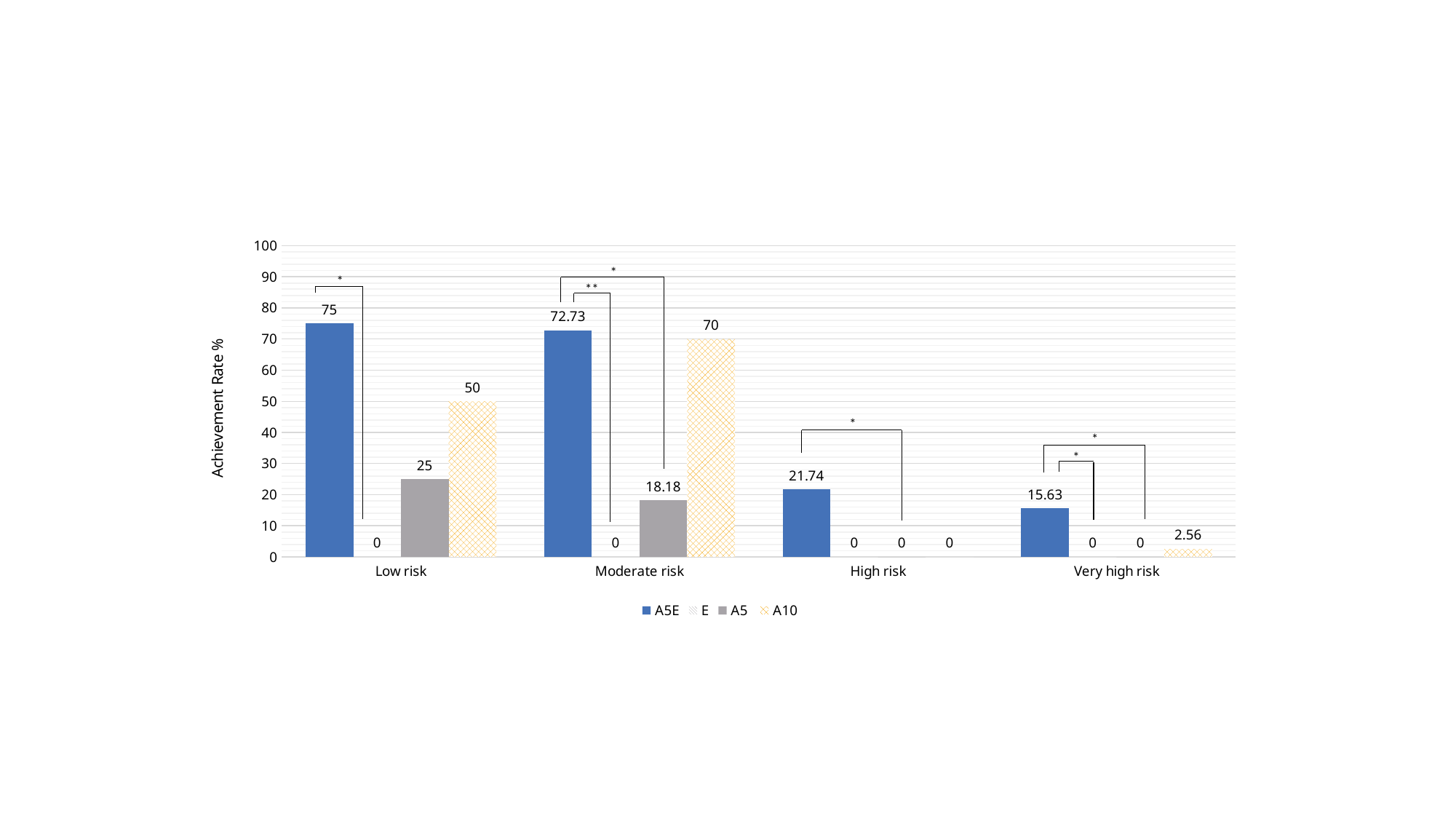

### Chart
| Category | A5E | E | A5 | A10 |
|---|---|---|---|---|
| Low risk | 75.0 | 0.0 | 25.0 | 50.0 |
| Moderate risk | 72.73 | 0.0 | 18.18 | 70.0 |
| High risk | 21.74 | 0.0 | 0.0 | 0.0 |
| Very high risk | 15.63 | 0.0 | 0.0 | 2.56 |*
*
**
*
*
*

Supplement: Supplementary file 7 [file medi-102-e36122-s007.pptx]
