# Supplementary material for: Effectiveness of low-intensity atorvastatin 5 mg and ezetimibe 10 mg combination therapy compared with moderate-intensity atorvastatin 10 mg monotherapy: A randomized, double-blinded, multi-center, phase III study
Source: Medicine (Baltimore). 2023 Nov 24;102(47):e36122. doi: 10.1097/MD.0000000000036122 (PMC10681377; doi:10.1097/MD.0000000000036122)
Supplement: Supplementary file 5 [file medi-102-e36122-s005.docx]

Supplementary Table 3. Changes in lipid parameters

|  | **Atorvastatin 5mg**  **Ezetimibe 10mg**  **(n=70)** | **Ezetimibe 10mg**  **(n=74)** | **Atorvastatin 5mg**  **(n=72)** | **Atorvastatin 10mg**  **(n=69)** |
| --- | --- | --- | --- | --- |
| **LDL-C (mg/dL)** |  |  |  |  |
| Baseline | 159.91 (38.94) | 156.34 (34.84) | 156.75 (32.71) | 150.51 (32.18) |
| Week 4 | 82.63 (26.32) | 127.12 (29.11) | 106.88 (30.39) | 91.52 (21.65) |
| Week 8 | 79.25 (21.10) | 125.94 (27.50) | 109.44 (30.70) | 93.78 (23.02) |
| % change from baseline |  |  |  |  |
| Week 4 | -48.36 (10.83) | -17.76 (12.76) | -30.45 (17.36) | -37.91 (14.92) |
| Week 8 | -49.16 (10.04) ^b), d), f)^ | -18.70 (13.49) | -27.89 (23.03) ^d), f)^ | -36.41 (19.04) |
| **HDL-C (mg/dL)** |  |  |  |  |
| Baseline | 47.09 (13.22) | 48.78 (15.40) | 47.85 (11.50) | 47.62 (10.61) |
| Week 4 | 51.02 (12.52) | 50.85 (15.65) | 50.39 (11.55) | 50.71 (11.88) |
| Week 8 | 50.21 (13.61) | 49.88 (16.33) | 49.92 (10.45) | 49.97 (11.59) |
| % change from baseline |  |  |  |  |
| Week 4 | 7.75 (15.11) | 5.43 (16.93) | 5.14 (14.80) | 7.25 (15.76) |
| Week 8 | 8.69 (14.41) ^a)^ | 2.49 (14.36) | 6.12 (15.24) | 6.16 (14.06) |
| **Non HDL-C (mg/dL)** |  |  |  |  |
| Baseline | 181.79 (39.34) | 176.15 (37.28) | 180.56 (36.61) | 172.33 (36.07) |
| Week 4 | 100.13 (29.15) | 143.75 (30.87) | 127.25 (32.57) | 109.81 (22.80) |
| Week 8 | 96.07 (23.68) | 143.90 (28.68) | 127.55 (32.46) | 110.87 (24.55) |
| % change from baseline |  |  |  |  |
| Week 4 | -44.82 (10.83) | -17.34 (12.73) | -28.24 (13.59) | -34.84 (14.30) |
| Week 8 | -46.00 (10.47) ^b), d), f)^ | -17.84 (10.70) | -28.01 (17.75) ^d), f)^ | -34.35 (17.47) |
| **LDL-C/HDL-C Ratio** |  |  |  |  |
| Baseline | 3.56 (1.02) | 3.44 (1.12) | 3.42 (0.96) | 3.28 (0.89) |
| Week 4 | 1.70 (0.66) | 2.68 (0.89) | 2.19 (0.66) | 1.89 (0.59) |
| Week 8 | 1.66 (0.54) | 2.73 (0.91) | 2.26 (0.68) | 1.96 (0.59) |
| % change from baseline |  |  |  |  |
| Week 4 | -51.57 (10.89) | -20.48 (15.96) | -33.54 (13.97) | -40.83 (17.31) |
| Week 8 | -52.72 (9.71) ^b), d), f)^ | -19.75 (14.62) | -31.75 (18.24) ^d), f)^ | -38.99 (20.32) |
| **TC (mg/dL)** |  |  |  |  |
| Baseline | 228.87 (43.02) | 224.93 (38.93) | 228.40 (37.03) | 219.96 (37.96) |
| Week 4 | 151.14 (31.92) | 194.60 (31.63) | 177.64 (34.23) | 160.52 (24.88) |
| Week 8 | 146.28 (27.95) | 193.78 (28.99) | 177.47 (34.47) | 160.84 (26.25) |
| % change from baseline |  |  |  |  |
| Week 4 | -33.92 (8.60) | -12.70 (10.43) | -21.37 (11.09) | -26.03 (11.18) |
| Week 8 | -34.79 (8.73) ^b), d), f)^ | -13.50 (9.14) | -21.18 (14.52) ^d), e)^ | -25.88 (13.28) |
| **TG (mg/dL)** |  |  |  |  |
| Baseline | 171.77 (69.39) | 158.07 (74.44) | 180.57 (81.38) | 173.39 (72.22) |
| Week 4 | 132.55 (68.19) | 137.71 (56.36) | 155.30 (110.37) | 134.97 (62.03) |
| Week 8 | 134.03 (67.49) | 152.07 (82.87) | 150.30 (67.49) | 139.97 (61.36) |
| % change from baseline |  |  |  |  |
| Week 4 | -13.64 (47.30) | -4.14 (34.38) | -6.04 (50.09) | -15.68 (36.28) |
| Week 8 | -16.70 (38.13) ^a)^ | 0.67 (36.81) | -11.31 (36.30) | -12.15 (39.53) |
| **ApoB (mg/dL)** |  |  |  |  |
| Baseline | 139.50 (30.36) | 133.70 (26.90) | 136.83 (27.25) | 131.03 (24.72) |
| Week 4 | 83.34 (22.94) | 113.12 (21.35) | 99.10 (23.57) | 87.65 (16.03) |
| Week 8 | 81.67 (19.32) | 112.72 (21.41) | 100.65 (23.67) | 88.45 (16.28) |
| % change from baseline |  |  |  |  |
| Week 4 | -39.68 (11.50) | -14.32 (10.84) | -26.17 (12.54) | -31.91 (12.52) |
| Week 8 | -40.34 (9.94) ^b), d), f)^ | -15.00 (11.67) | -25.07 (15.49) ^d), f)^ | -31.52 (14.41) |
| **ApoA1 (mg/dL)** |  |  |  |  |
| Baseline | 148.39 (29.81) | 147.92 (29.60) | 149.56 (22.31) | 149.83 (25.91) |
| Week 4 | 154.78 (25.99) | 151.81 (33.81) | 155.57 (25.48) | 153.25 (24.99) |
| Week 8 | 154.66 (30.01) | 149.99 (32.51) | 153.08 (21.25) | 152.67 (25.64) |
| % change from baseline |  |  |  |  |
| Week 4 | 4.12 (11.53) | 2.89 (12.78) | 3.95 (11.29) | 3.09 (12.15) |
| Week 8 | 5.67 (14.78) | 1.41 (10.65) | 3.31 (11.24) | 3.00 (11.86) |

HDL-C = high-density lipoprotein cholesterol; LDL-C = low-density lipoprotein cholesterol; TC = total cholesterol; TG = triglyceride

p-value from MMRM (Mixed effect Models for Repeated Measures)

^a^ P:<0.05 vs Ezetimibe 10mg

^b^ P:<0.01 vs Ezetimibe 10mg

^c^ P:<0.05 vs Atorvastatin 5mg

^d^ P:<0.01 vs Atorvastatin 5mg

^e^ P:<0.05 vs Atorvastatin 10mg

^f^ P:<0.01 vs Atorvastatin 10mg
