# Supplementary material for: Effectiveness of low-intensity atorvastatin 5 mg and ezetimibe 10 mg combination therapy compared with moderate-intensity atorvastatin 10 mg monotherapy: A randomized, double-blinded, multi-center, phase III study
Source: Medicine (Baltimore). 2023 Nov 24;102(47):e36122. doi: 10.1097/MD.0000000000036122 (PMC10681377; doi:10.1097/MD.0000000000036122)
Supplement: Supplementary file 8 [file medi-102-e36122-s008.pptx]

## Slide 1
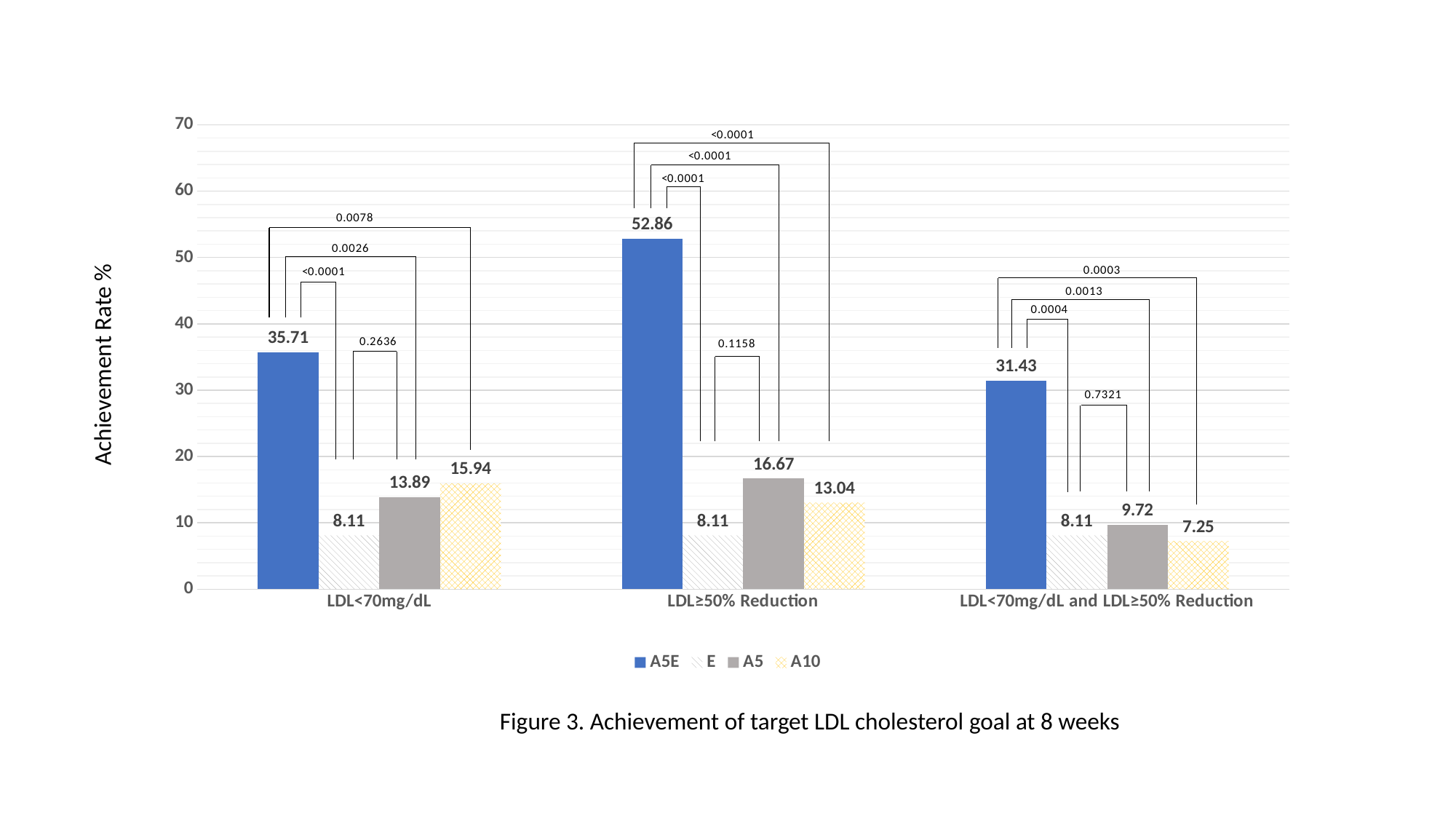

### Chart
| Category | A5E | E | A5 | A10 |
|---|---|---|---|---|
| LDL<70mg/dL | 35.71 | 8.11 | 13.89 | 15.94 |
| LDL≥50% Reduction | 52.86 | 8.11 | 16.67 | 13.04 |
| LDL<70mg/dL and LDL≥50% Reduction | 31.43 | 8.11 | 9.72 | 7.25 | Achievement Rate %
Figure 3. Achievement of target LDL cholesterol goal at 8 weeks
